# Prognostic value of increased KPNA2 expression in some solid tumors: A systematic review and meta-analysis

**DOI:** 10.18632/oncotarget.13863

**Published:** 2016-12-10

**Authors:** Li-Na Zhou, Yue Tan, Ping Li, Ping Zeng, Min-Bin Chen, Ye Tian, Ya-Qun Zhu

**Affiliations:** ^1^ Department of Radiotherapy and Oncology, the Second Affiliated Hospital of Soochow University, Institute of Radiotherapy & Oncology, Soochow University, Suzhou, Jiangsu 215004, China; ^2^ Department of Radiotherapy and Oncology, Kunshan First People's Hospital Affiliated to Jiangsu University, Kunshan 215300, Jiangsu Province, China

**Keywords:** KPNA2, tumor, prognosis, overall survival

## Abstract

**Background:**

Karyopherin α2 (KPNA2), a member of the Karyopherin α family, has recently been reported to play an important role in tumor progression. However, the association between KPNA2 expression and prognosis in cancer remains controversial. So we performed this meta-analysis to evaluate whether expression of KPNA2 was associated with prognosis in patients with solid tumor.

**Methods/Findings:**

24 published eligible studies, including 6164 cases, were identified and included in this meta-analysis through searching of PubMed, EMBASE and Web of Science. We found that KPNA2 expression was an independent predictor for the prognosis of solid tumor with primary outcome (overall survival [OS]: pooled HR=1.767, 95% CI=1.503-2.077, *P*<0.001) and secondary outcomes (time to recurrence [TTR], recurrence free survival [RFS] and progression free survival [PFS]). However, the association between KPNA2 overexpression and disease free survival [DFS] in solid tumors was not significant (pooled HR=1.653, 95% CI=0.903-3.029, *P*=0.104). Furthermore, the subgroup analysis revealed that KPNA2 overexpression was associated with poor OS in East-Asian patients and European patients, as well as patients with gastric and colorectal cancer.

**Conclusion:**

KPNA2 expression may be a useful prognostic biomarker to monitor cancer prognosis. Further prospective studies with larger sample sizes are required to confirm our findings.

## INTRODUCTION

Cancer is a main public health problem worldwide. Although, the overall mortality declined over the past two decades, cancer remains one of the main contributor of human mortality [1]. Dysfunction of cellular transport machinery is often observed in caner. The shuttling of proteins between the cytoplasm and the nucleus is mediated by karyopherins. Karyopherin α2 (KPNA2) is one of seven described members of the karyopherin α family, which is also known as importin α-1 or RAG cohort 1. KPNA2 weighs around 58 kDa and is composed of a N-terminal hydrophilicimportinb-binding domain, a central hydrophobic region, and a short acidic C-terminus [2]. KPNA2 may participate in carcinogenesis through regulating the subcellular translocation of cancer-associated cargo proteins [3]. KPNA2 overexpression was shown to promote G1/S cell cycle transition via upregulating c-Myc. KPNA2 could also enhance transcriptional activity of c-Myc, activate Akt, and suppress FOXO3a in various cancer cells. Meanwhile, downregulation of cyclin-dependent kinase (CDK) inhibitor p21 and p27, as well as upregulation of CDK regulator cyclin D1 were seen in KPNA2-over-expresssed cells [4]. Forced expression of KPNA2 could increase proliferation of breast cancer cells [5]. On the other hand, knockdown of KPNA2 was shown to inhibit proliferation of cancer cells derived from lung [6], liver [7] and prostate cancer [8]. Growth evidences have also proposed the potential role of KPNA2 in multiple cancerous behaviors, including cell proliferation, differentiation, cell-matrix adhesion, colony formation and migration [5].

Existing evidences have shown that KPNA2 was over-expressed in multiple malignancies [9–11]. Meanwhile, it has been suggest that elevated KPNA2 could be associated with poor prognosis in a variety of solid tumors, including colorectal cancer [11–13], breast cancer [14–17], gastric cancer [10, 18, 19] and hepatocellular carcinoma [20, 21]. Intriguingly, it was reported that low cytoplasmic and nuclear KPNA2 expression may also predict an adverse outcome in radiotherapy-treated head and neck squamous cell cancer [22]. The results of those individual studies were controversial. Therefore, we conducted this meta-analysis to overcome the limitation of the single study.

## RESULTS

### Demographic characteristics

**Figure 1:**
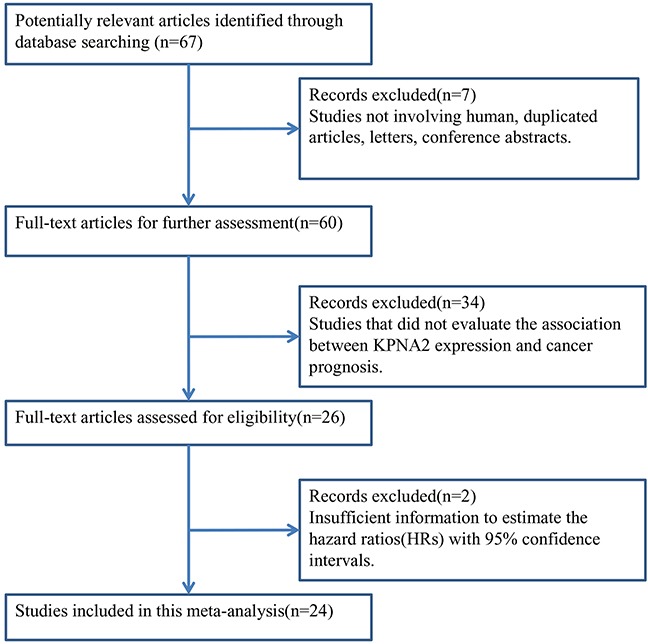
The flow chart of the selection process in our meta-analysis

Using the described combinations of key terms, a total of 67 articles were retrieved from a literature search of PubMed, Embase and Web of Science databases. As displayed in the search flow diagram (Figure 1) and updated Prisma checklist (Supplementary Table S1), 24 articles published from 2006-2016, which reported at least one of the mentioned outcomes, were included in this meta-analysis [4, 8, 10–31].

All studies were graded by Newcastle-Ottawa Scale (NOS) (Supplementary Table S2). The NOS scores ranged from 7 to 9, showing that the methodological quality was high. The main features of these eligible articles were listed in Table 1.

**Table 1:** Characteristics of studies included in the meta-analysis

First author	Year	Country	Case	Cancer type	Detection	Provided information on cutoff value	Outcome endpoints	NOS score
Tsai MM [18]	2016	Taiwan	77	gastric cancer	IHC	score ≥ 40	OS	9
Zhang Y [12]	2015	China	195	colon cancer	IHC	score ≥3(range of 0-7)	OS,DFS	8
Takada T [13]	2015	Japan	135	colorectal cancer	IHC	Low = score 0-4; high = score 6, 9	OS	8
Alshareeda AT [14]	2015	UK	1494	breast cancer	IHC	negative/low<35, positive≥35H-score(range of 0-300)	OS	9
Shi B [29]	2015	China	176	upper tract urothelial carcinoma	IHC	strong nuclear staining in at least 10%	OS,DFS	9
Erben PB [22]	2015	Germany	225	head and neck squamous cell cancer	IHC	the percentage of positive stained nuclei >15%(median)	DFS	8
Hu ZY [20]	2014	China	314	hepatocellular carcinoma	IHC	nucleus staining in more than 5% cells	OS,RFS	7
Jiang P [21]	2014	China	221	hepatocellular carcinoma	IHC	extent≥5% (range from 0 to 100%)	OS,TTR	9
Gousias K [24]	2014	Germany	108	meningiomas	IHC	the percentage of moderately or strongly immunopositive cell nuclei ≥5% (median)	PFS	9
Ikenberg K [26]	2014	Switzerland	527	endometrial cancer	IHC	strong nuclear staining in at least 10% of nuclei	OS	9
Huang L [4]	2013	China	191	epithelial ovarian carcinoma	qRT-PCR	expression level of KPNA2>3.52	OS.RFS	8
Altan B [19]	2013	Japan	179	gastric cancer	IHC	Low = score 0-3; high = score 4, 6, 9	OS	9
Rachidi SM [11]	2013	USA	54	colon cancer	IHC	nuclear staining intensity score > 3	OS	8
Li C [10]	2013	China	142	gastric cancer	IHC	score ≥ 4(range of 0-9)	OS	8
He L [25]	2012	China	90	ovarian malignant germ cell tumor	IHC	score ≥ 2.5(range of 0-12)	OS,DFS	7
Gousias K (a) [23]^#^	2012	Germany	94	Astrocytomas	IHC	≥5% nuclear immunoreactivity	OS,PFS	9
Gousias K (b) [23]^#^	2012	Germany	47	Glioblastomas	IHC	≥10% nuclear immunoreactivity	OS,PFS	9
Mortezavi A (a) [8]^#^	2011	Switzerland	341	prostate cancer	IHC	Nuclear KPNA2 immunoreactivity>0%	RFS	9
Mortezavi A (b) [8]^#^	2011	Switzerland	237	prostate cancer	IHC	Nuclear KPNA2 immunoreactivity>0%	RFS	9
Jensen JB [27]	2011	Denmark	377	bladder cancer	IHC	nuclear staining of ≥10% of the carcinoma cells	OS,RFS	8
Zheng M [31]	2010	China	102	epithelial ovarian carcinoma	IHC	scores of (++) and (+++) were recorded as positive	OS	9
Sakai M [28]	2010	Japan	116	Esophageal Squamous Cell Carcinoma	IHC	KPNA2 LI(labeling index) ≥10.7%(range 0-44.3%)	OS	8
Gluz O [15]	2008	Germany	191	breast cancer	IHC	nuclear expression >10% of nuclei	OS	8
Dankof A [16]	2007	Germany	83	breast cancer	IHC	nuclear expression >10% of nuclei	DFS	9
Dahl E [17]	2006	Germany	272	breast cancer	IHC	nuclear expression≥10% of nuclei	OS	9
Winnepenninckx V [30]	2006	Belgium	176	melanoma	IHC	>average expression value	OS	9

IHC : Immunohistochemistry; qRT-PCR:Quantitative Real Time Polymerase Chain Reaction;NOS: Newcastle-Ottawa Scale; DFS: disease free survival; TTR: time to recurrence ; RFS: recurrence free survival; PFS: progression free survival.

# There were two parts of data(a and b)in each of the studies of Gousias K and Mortezavi A.

Together, the 24 eligible studies provided a sample size of 6164 patients, which were utilized to evaluate the relationship between KPNA2 expression and solid tumors' prognosis. The median sample-size was 177, with a wide range from 47 to 1494. Among all cohorts, China (n = 8) was the major source region, followed by Germany (n = 7) and Japan (n = 3). As for the cancer type, four studies evaluated breast cancer, three studies evaluated colorectal cancer, three studies evaluated gastric cancer, two studies evaluated hepatocellular carcinoma, two studies evaluated epithelial ovarian carcinoma, one study evaluated prostate cancer, one study evaluated bladder cancer, one study evaluated esophageal squamous cell carcinoma, one study evaluated endometrial cancer, one study evaluated melanoma, one study evaluated ovarian malignant germ cell tumor (OMGCT), one study evaluated upper tract urothelial carcinoma, one study evaluated meningiomas, one study evaluated anaplastic gliomas, one study evaluated astrocytomas. As for the survival outcomes, among 24 eligible studies, twenty of them focused on primary outcome (OS), thirteen studies focused on secondary outcomes (5 for DFS, 4 for RFS, 2 for PFS and 1 for TTR) (Table 1).

### Evidence synthesis

**Figure 2:**
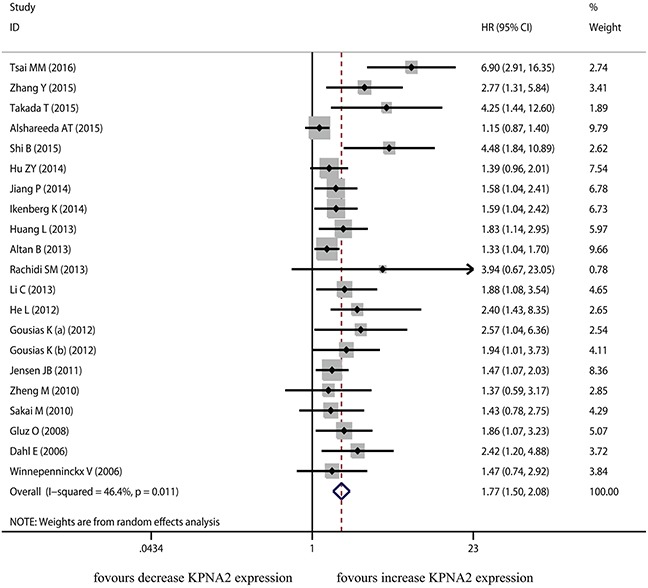
The correlation between KPNA2 expression and overall survival in solid tumor

The current meta-analysis was based on primary outcome (OS) and secondary outcomes (TTR, RFS, PFS and DFS). Twenty studies were included in the meta-analysis of OS. A random-effects model was applied to calculate the pooled hazard ratio (HR) and 95% confidence interval (CI). The heterogeneity test reported the *P* value of 0.011 and *I*^2^ values of 46.4%. These results showed an evidence of significant association between KPNA2 overexpression and poor OS (pooled HR=1.767, 95% CI=1.503-2.077, *P*<0.001) (Figure 2).

**Figure 3:**
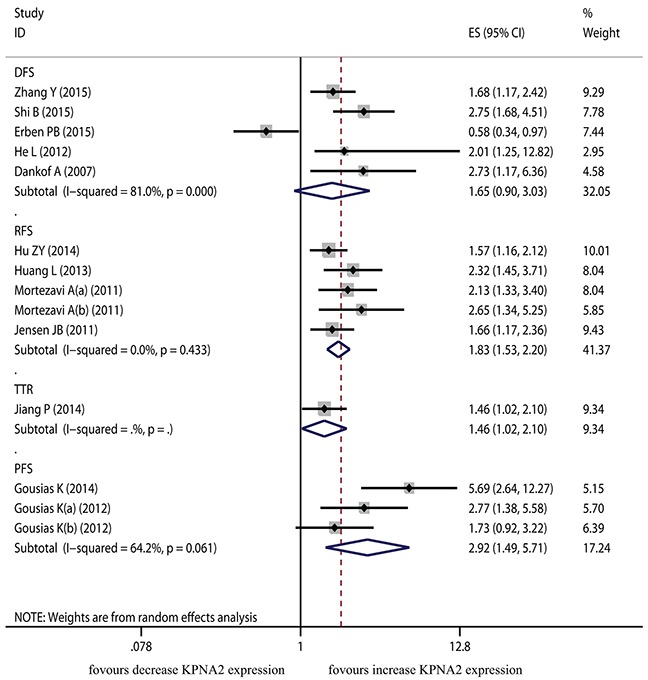
The correlation between KPNA2 expression and time to tumor progression in solid tumor

A random-effects model was utilized to calculate the pooled HR and 95% CI in 5 studies which focused on DFS, as the heterogeneity test reported the *P* value <0.001 and *I*^2^ value of 81.0%. The pooled result showed the association between KPNA2 overexpression and DFS was not significant (pooled HR=1.653, 95% CI=0.903-3.029, *P*=0.104) (Figure 3).

The TTR was derived from only one dataset and showed significant association with KPNA2 overexpression (HR=1.464, 95% CI=1.023-2.096, *P*=0.037). The pooled results from five datasets for RFS and three datasets for PFS indicated that KPNA2 overexpression was associated with poor RFS and poor PFS (HR=1.835, 95% CI=1.530-2.200, *P*<0.001; HR=2.921, 95% CI=1.493-5.715, *P*=0.002, respectively).

To explore the source of heterogeneity, subgroup analyses were conducted by origin of patients and cancer types. The results of subgroup analysis were presented in Table 2. In the subgroup stratified by origin of patients, the pooled HR was 1.962 (95% CI = 1.525-2.525, *P*<0.001) in East-Asian populations from 12 included studies. The pooled HR was 1.562 (95% CI = 1.407-1.734, *P*<0.001) for European group from the other 8 studies. Both of the two overall outcomes indicated the significant relationship between KPNA2 overexpression and poor OS. For the analysis stratified by cancer type, significant association between KPNA2 overexpression and poor OS was observed in patients with gastric cancer (HR = 2.353, 95% CI = 1.048-5.284, *P* = 0.038) and colorectal cancer (HR = 3.252, 95% CI = 1.82-5.811, *P*<0.001), but not in patients with breast cancer (HR = 1.588, 95% CI = 0.996-2.531, *P* = 0.052).

**Table 2:** Hazard ratio for the association between KPNA2 expression and solid tumor prognosis

Analysis		N	References			Heterogeneity	
				HR(95% CI)	*P*	*I^2^*(%)	*Ph*
All Studies							
OS		20	[4, 10–15, 17–21, 23, 25–31]	1.767(1.503-2.077)	<0.001^#^	46.4	0.011
TTR		1	[21]	1.464(1.023-2.096)	0.037	-	-
RFS		4	[4, 8, 20, 27]	1.835(1.530-2.200)	<0.001^#^	0.0	0.433
PFS		2	[23, 24]	2.921(1.493-5.715)	0.002	64.2	0.061
DFS		5	[12, 16, 22, 25, 29]	1.653(0.903-3.029)	0.104	81.0	<0.001^#^
Origin of patients							
East-asian	OS	12	[4, 10, 12, 13, 18–21, 25, 28, 29, 31]	1.962(1.525-2.525)	<0.001^#^	56.7	0.008
European	OS	8	[11, 14, 15, 17, 23, 26, 27, 30]	1.562(1.407-1.734)	<0.001^*^	21.6	0.251
Cancer type							
gastric cancer	OS	3	[10, 18, 19]	2.353(1.408-5.284)	0.038^#^	85.1	0.001
breast cancer	OS	3	[14, 15, 17]	1.588(0.996-2.531)	0.052^#^	64.5	0.060
colorectal cancer	OS	3	[11–13]	3.252(1.82-5.811)	<0.001^*^	0.0	0.796

N:number of studies; *Ph*: *p* value of Q-test for heterogeneity;

* The pooled HR was calculated using a fixed-effects model (the Mantel–Haenszel method) according to the heterogeneity;

# The pooled HR was calculated using a random-effects model (the DerSimonian and Laird method) according to the heterogeneity; Subgroup analysis was performed when there were at least three studies in each subgroup.

### Publication bias and sensitivity analysis

**Figure 4:**
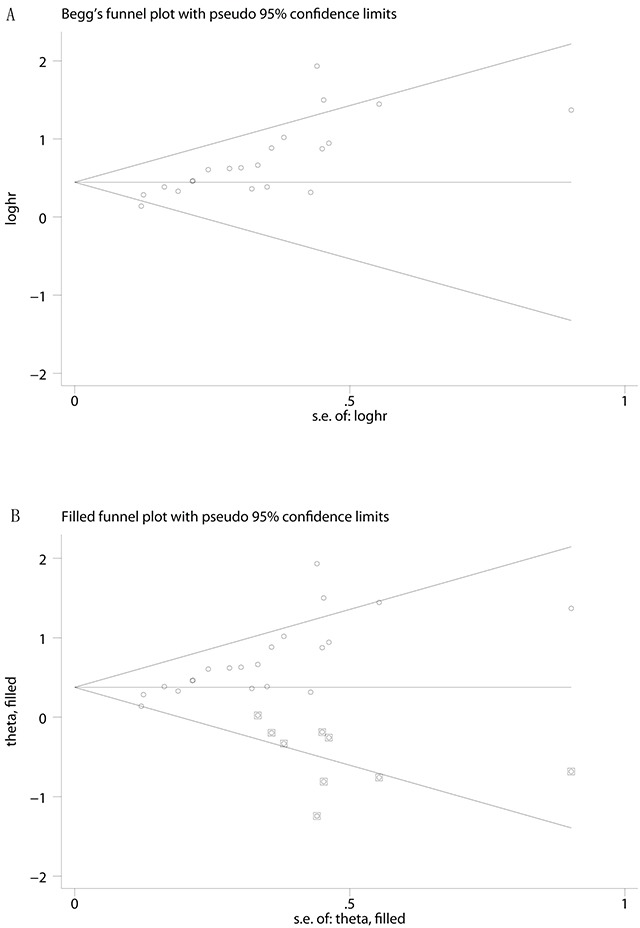
Begg's funnel plots for the studies involved in the meta-analysis of KPNA2 expression and the prognosis of patients with solid tumors **A.** Publication bias influence on the overall effect was assessed by the Duval and Tweedie's trim and fill method Duval and Tweedie's trim and fill method **B.** Abbreviations: loghr, logarithm of hazard ratios; s.e., standard error.

As the amount of datasets for meta-analysis of secondary outcomes (TTR/PFS/RFS/DFS) was fewer, this meta-analysis only evaluated the publication bias for the primary outcome (OS). Begg's funnel plot and Egger's test were applied to evaluate the publication bias of the literatures. The funnel plot was asymmetrical. The *P* value calculated from Egger's test pointed out the presence of publication bias (*P*<0.001) among these studies (Figure 4A). Therefore, we performed trim and fill method to make pooled HR more reliable (Figure 4B), and the *P* value was less than 0.01(data not shown).

**Figure 5:**
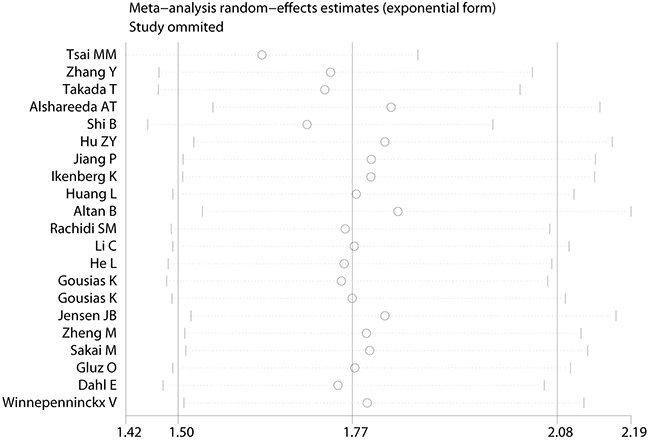
Sensitivity analysis of the meta-analysis (Overall survival)

Furthermore, sensitivity analysis was conducted to assess the influence of individual study on the summary effects for the OS. None of the each single study dominated this meta-analysis, and the removal of each study had no significant effect on the overall conclusion (Figure 5). Removal of study using Quantitative Real Time Polymerase Chain Reaction (qRT-PCR) to assess the expression of KPNA2 obtained similar results of OS (HR =1.773, 95% CI = 1.495-2.102, *P*<0.001, *I*^2^ = 48.4%). There were 4 studies with the number of cases less than 100, elimination of these studies had no substantial impact on the outcome of OS (HR = 1.583, 95% CI = 1.372-1.826, *P*<0.001, *I*^2^ = 30.7%).

## DISCUSSION

Many studies have indicated that the aberrant expression of KPNA2 is closely associated with tumor genesis and cancer progression [8–15, 17, 19, 21, 25, 27, 29, 31]. KPNA2 is shown to participate in the translocation of cancer-associated cargo proteins, such as Chk2 [32], BRCA1 [33], NBS1 [2] and many others. In addition, clinical studies have investigated the potential prognostic value of KPNA2. Most of these studies, however, include only limited number of patients, and the results remain inconclusive. To the best of our knowledge, the current meta-analysis was the first systematic evaluation of the literatures studying tumor prognosis and KPNA2 expression.

We evaluated survival data from 6,164 solid tumor patients from 24 different studies. Our results suggest that the increased expression of KPNA2 is indeed a poor prognostic marker for solid tumors in primary outcome (OS pooled HR=1.767, 95% CI=1.503-2.077, *P*<0.001) and secondary outcomes (TTR/RFS/PFS).

There are several important implications from results of this meta-analysis. First, KPNA2 might serve as a reliable prognostic marker for solid tumors. In this meta-analysis, we included fifteen different cancer types, including breast cancer [14–17], colorectal cancer [11–13], gastric cancer [10, 18, 19], prostate cancer [8], hepatocellular carcinoma [20, 21], epithelial ovarian carcinoma [4, 31], bladder cancer [27], esophageal squamous cell carcinoma [28], endometrial cancer [26], melanoma [30], OMGCT [25], upper tract urothelial carcinoma [29], meningioma [24], astrocytoma [23], head and neck squamous cell cancer [22]. The overall pooled results from these cancer types indicated that elevated KPNA2 expression was associated with patients' poor OS, TTR, RFS and PFS. We therefore propose that high KPNA2 expression may have similar prognostic value for other types of tumor. Second, we demonstrated that KPNA2 overexpression correlated with poor OS in East-Asian population and European population. Different genetic background has no significant effect on the results. Finally, when data was stratified according to cancer type, the results showed the prognostic value of KPNA2 overexpression for OS was significant in gastric cancer and colorectal cancer. In breast cancer, KPNA2 overexpression was associated with poor outcome, but lack of statistical significance. The limited sample size from certain cancer types might have also been statistically insufficient to detect any small effect.

Apart from the inspiring outcomes, there are several potential limitations of this meta-analysis, which should be considered to interpret the outcomes. First, this meta-analysis only enrolled fully published studies in PubMed or EMBASE, yet conference abstracts and studies without enough data were excluded. Second, studies were more likely to be published if they have positive results than negative results. Our analysis detected some publication bias, however meta-analyses with and without the “trim and fill” method did not produce different conclusions. Third, although most of the studies detected the KPNA2 expression by IHC, the antibody concentration and the cutoff value varied across different studies, which might cause some biases in pooled analysis. Fourth, the number of patients of certain published studies, and the number of published studies of one single cancer types may not be sufficient enough for a comprehensive analysis, and our results should be extended to other specific tumor types cautiously. Therefore, our estimate of the association between increased KPNA2 and poor prognosis could possibly be overestimated.

## CONCLUSION

In conclusion, our results demonstrate that overexpression of KPNA2 is associated with poor prognosis in various tumors. KPNA2 might be a promising prognostic biomarker and a potential therapeutic target for solid tumors.

## MATERIALS AND METHODS

### Publication search

PubMed, Embase, and Web of Science databases were searched (up to June 23, 2016) using the search terms: “KPNA2[All Fields] AND (“neoplasms”[MeSH Terms] OR “neoplasms”[All Fields] OR “cancer”[All Fields])) AND (“prognosis”[MeSH Terms] OR “prognosis”[All Fields]) OR (“mortality”[Subheading] OR “mortality”[All Fields] OR “survival”[All Fields] OR “survival”[MeSH Terms]) OR predict[All Fields] OR outcome[All Fields] OR (“life”[MeSH Terms] OR “life”[All Fields] OR “alive”[All Fields])”. All potentially eligible studies were retrieved and their bibliographies were carefully scanned to identify other eligible studies. Additional studies were identified by a hand search of the references cited in the original studies. When multiple studies of the same patient population were identified, we only included the published report with the largest sample size. Additionally, updated Prisma checklist and flow chart were used to present the search strategy.

### Inclusion and exclusion criteria

Studies included in this meta-analysis had to meet all the following criteria: (a) evaluation of KPNA2 expression for predicting cancer prognosis; (b) studies reporting survival data; (c) studies provided enough data for individual HRs and 95% CIs to be extracted or calculated; and (d) studies published in English. The exclusion criteria were as follows: 1) review articles, case reports, letters to the editor, conference abstracts, experimental studies and commentary articles; 2) over-lapping or double data; 3) inadequate survival data for further quantification; and 4) the follow-up duration was shorter than 3 years.

### Data extraction and methodological quality assessment

This meta-analysis of KPNA2 expression was based on following outcome endpoints: primary outcome (OS) and secondary outcomes [time to recurrence (TTR), recurrence free survival (RFS), progression free survival (PFS) and disease free survival (DFS)]. According to the inclusion and exclusion criteria above, the following items were extracted from each study: the first author's surname, year of publication, country of origin, number of cases, type of cancer, method of detection, score for KPNA2 assessment and cut-off value to determine KPNA2 positivity, Hazard ratio (HR) of KPNA2 expression for OS, TTR, RFS, PFS and DFS with the 95% CI and P-value. If only Kaplan-Meier curves were presented in the studies, we utilized Engauge Digitizer version 4.3 to obtain the survival data, and Tierney's method to calculate the HRs and 95%Cis [34]. Subgroup analysis was performed when there were at least three studies in each subgroup. Data from all eligible publications were extracted carefully and independently by two of the authors. Any disagreements between the researchers were resolved through extracting data from the original article independently by the third author, and any discrepancy was resolved by consensus review.

The methodological quality assessment of each study was performed using the Newcastle–Ottawa Scale(NOS) [35], which scored studies with 9 items including the selection of the patient population, study comparability, outcome of interest, follow-up et al. Studies with an NOS score ≥6 were considered as high-quality ones.

### Statistical analysis

In order to evaluate the relationship between KPNA2 expression and solid tumor prognosis, we applied HRs with their corresponding 95% CIs from each eligible paper to calculate the pooled HR for outcome endpoints (OS, DFS, RFS, PFS and TTR). The overall HR was >1, and the 95% CI did not overlap in the forest plot, suggesting a poor prognosis in patients with high expression of KPNA2. Heterogeneity assumption among the included studies was checked using Cochran's Q test and Higgins's *I*^2^ statistic [36], *P* value >0.10 and *I*^2^ <50% suggested a lack of heterogeneity among studies. In absence of heterogeneity, a fixed-effects model was applied. Otherwise, the random-effects model was employed [37]. Funnel plots and the Egger's test were utilized to evaluate the possible publication bias [38]. If a publication bias did exist, its influence on the overall effect was assessed by the Duval and Tweedie's trim and fill method [39].

Sensitivity analysis was also performed by omitting each study or specific studies to find potential outliers. All statistical analyses were performed via Stata 14.0 (StataCorp, College Station, TX). All *P* values for comparisons were two-sided and statistical significance was defined as *P*<0.05, except those for heterogeneity.

## SUPPLEMENTARY TABLES






