# Supplementary material for: Prognostic value of increased KPNA2 expression in some solid tumors: A systematic review and meta-analysis
Source: Oncotarget. 2016 Dec 10;8(1):303–14. doi: 10.18632/oncotarget.13863 (PMC5352121; doi:10.18632/oncotarget.13863)
Supplement: Supplementary file 1 [file oncotarget-08-303-s001.pdf]

Prognostic value of increased KPNA2 expression in some solid tumors: A systematic review and Meta-Analysis

SUPPLEMENTARY TABLES

Supplementary Table S1: Prisma checklist.

See Supplementary File 1

Supplementary Table S2: Additional main features of these eligible articles.

See Supplementary File 2
